# A Misdiagnosed Case of Malignant Melanoma in an Infected Nail: A Case Report

**DOI:** 10.7759/cureus.60236

**Published:** 2024-05-13

**Authors:** Priya B Chatterjee, K. M. Hiwale, Shreya Ghosh

**Affiliations:** 1 Pathology, Jawaharlal Nehru Medical College, Datta Meghe Institute of Higher Education and Research, Wardha, IND

**Keywords:** nail bed tumors, melanoma, malignant melanoma, acral lentiginous melanoma, subungual melanoma

## Abstract

Subungual melanoma is associated with the highest mortality among all skin cancers and is strongly linked to acquired mutations caused by exposure to ultraviolet radiation in sunlight. The commonest sites of occurrence are the great toe and thumb. Diagnosis of melanoma often becomes a challenge as it is difficult to differentiate it from other pigmented disorders. A histopathological evaluation of the lesion with adequate nail matrix biopsy can address the diagnostic dilemma. Additionally, an early diagnosis of melanoma is critical as once detected early, it is often treatable. We present a case of a 72-year-old diabetic male patient with a pigmented lesion over the right great toe. In view of the patient’s age and history of diabetes, the initial presentation was mistaken as onychomycosis which created a diagnostic dilemma. Hence, we present this case to shed light upon the fact that these lesions can mimic several other benign conditions like fungal melanonychia, lentigo, and subungual hemorrhage. To avoid misdiagnosis and subsequent delay in management, early clinical, dermoscopic, and very pertinently, histopathological and radiological co-relations are extremely important.

## Introduction

Subungual melanoma is a rare variant of acral lentiginous melanoma, a malignant melanoma arising from palms and soles. Subungual melanoma originates from the nail matrix [1]. The subungual form comprises 0.7-3.5% of all cutaneous melanomas, accounting for 10-23% in Asians, 25% in Africa-Americans, and 0.18-2.8% in Europeans [1,2]. There is no specific data available from India regarding the disease incidence to date. The common risk factors for subungual melanoma are trauma, chronic inflammation, and mechanical stress with an average age of diagnosis being 60-70 years of age [1,3,4]. With varied clinical presentations and low survival rates, subungual melanoma is a disease associated with significant mortality. A good clinical, dermoscopic examination, such as periungual multicolor pigmentation, broadened width of asymmetric bands with irregular pattern, and finally, a good nail matrix biopsy with histopathological examination, can help in timely and early diagnosis of this condition, which in turn is associated with a good prognosis. The complete removal of the tumor by excision or amputation at an early stage is curative, while invasive disease increases the risk of distant metastasis [2,5].

## Case presentation

A 72-year-old male of Indian ethnicity, with a history of diabetes for 10 years, presented to the dermatology outpatient department with a pigmented lesion over his right great toe for the past four years. This pigmentation was progressive in nature, associated with mild loss of sensation which developed post-trauma. The discoloration progressed to thickening, splitting, and full destruction of the nail. The patient provided a past history of having consulted a general practitioner three years back and had received several courses of topical antibiotics and antifungals for a few months, without improvement in the lesion. There was no significant family history of skin or nail cancer.

**Figure 1:**
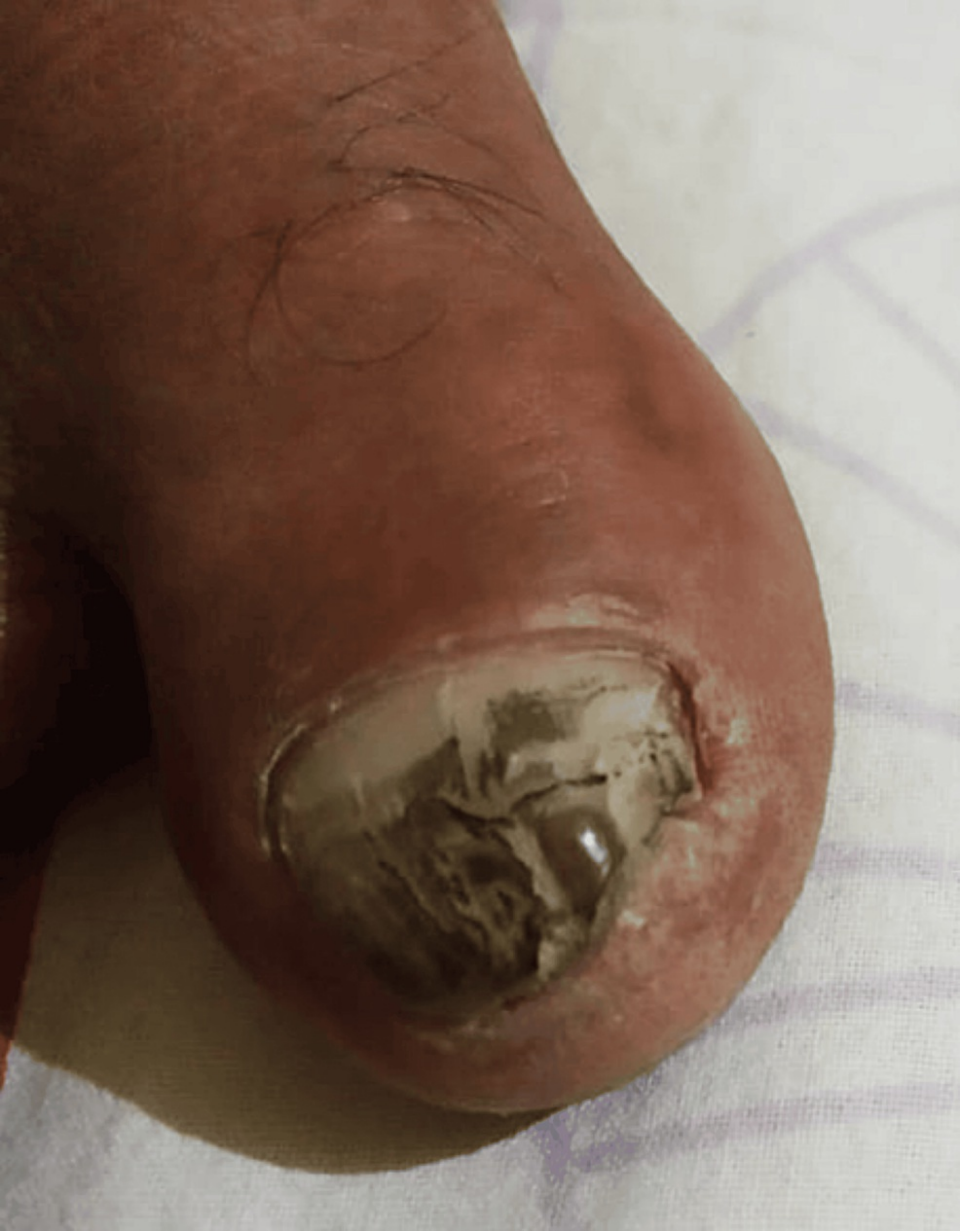
Image of the right great toe Blackish discoloration of the right great toe's nail bed with ill-defined borders appearing in the form of bands

The examination of the right great toe revealed a blackish discoloration of the nail bed with ill-defined borders that appeared in the form of bands, with Hutchinson’s sign, without any tenderness, as seen in Figure 1. The remaining fingernails appeared normal.

**Figure 2:**
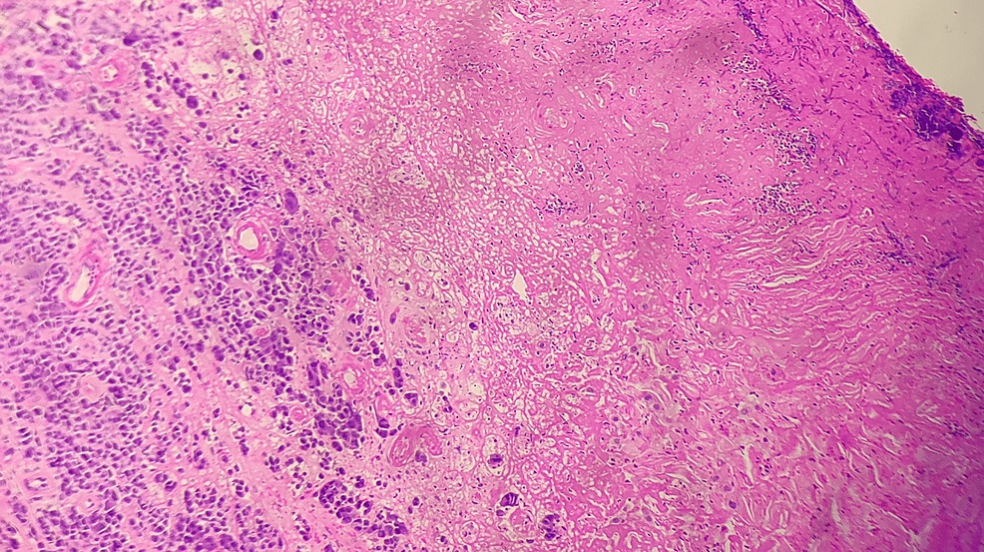
Scanner view of H&E stained slide of the infected nail H&E stained slide of an infected nail shows ulcerated squamous epithelium with ribbon-like hyphae and underlying subepithelial tissue shows infiltration by a high-grade malignant tumor arranged in nests, sheets, and as single cells H&E: Hematoxylin and eosin

A dermoscopic examination performed by the dermatologist revealed a pigmentation appearing in the form of bands involving more than two-thirds of the nail plate. The color noted was brown, with irregularly notched borders. Multiple biopsy specimens were taken from the nail bed and nail matrix and sent to the histopathology section in 10% buffered formalin. A gross examination of the received specimens revealed multiple, irregular, and greyish-brown tissue bits, with partially attached nails, aggregating 1.2 × 1 cm. Zones of white hypopigmented areas were also identified. Staining with hematoxylin and eosin (H&E) showed ulcerated squamous epithelium, with the underlying subepithelial tissue showing infiltration by a high-grade malignant tumor arranged in nests, sheets, and as infiltrating single cells, as seen in Figure 2. The individual cells had pleomorphic nuclei, prominent nucleoli, and a moderate amount of amphophilic cytoplasm. A mitosis was seen. The intracytoplasmic pigment was noted with large areas of necrosis. Also, in the epidermis, ribbon-like hyphae were noted, giving evidence of fungal invasion in association with the tumor. The Breslow depth of invasion was 2 mm.

**Figure 3:**
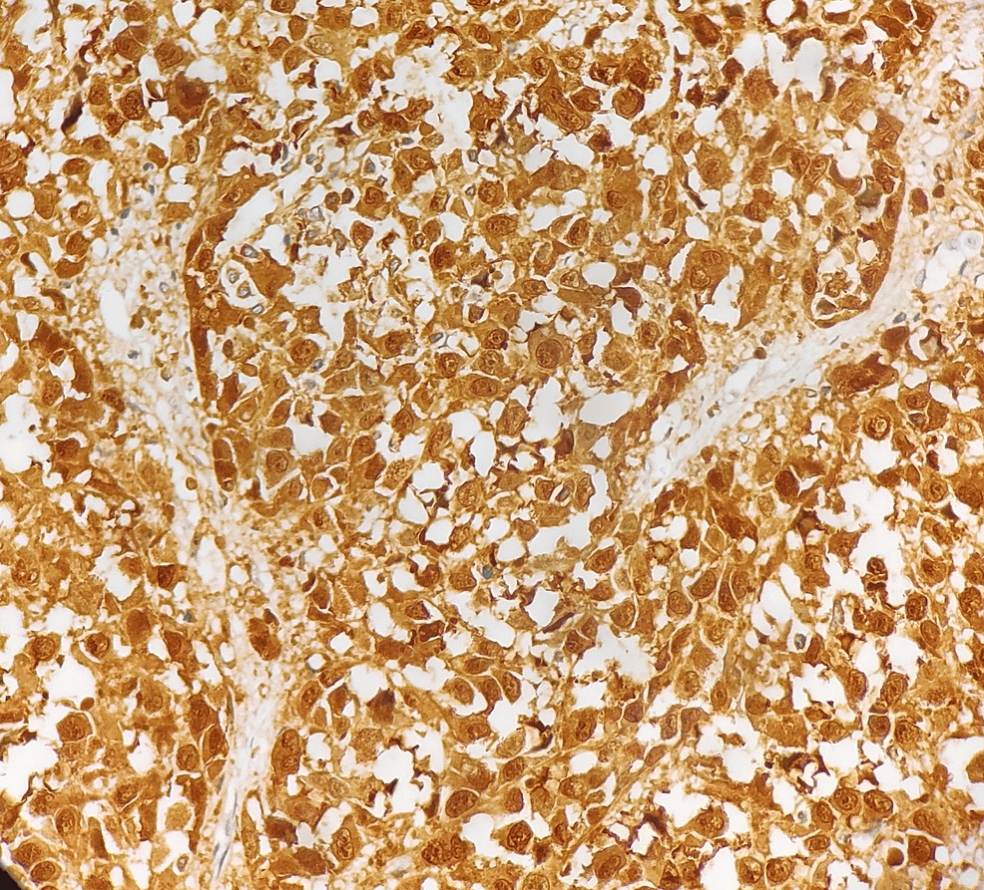
Immunohistochemistry-S100 protein of the biopsy specimen from the right great toe The 40x image of the immunohistochemistry-S100 protein of the right great toe biopsy specimen is strongly positive

The immunohistochemistry showed tumor cells that were immune-positive for S100 protein, as seen in Figure 3. They were also positive for melanoma antigen A (Melan-A) and negative for pan-cytokeratin. The histopathological features were confirmatory of subungual melanoma of the right-sided great toe, with no evidence of lymphovascular or perineural invasion. Radiological investigations like computed tomography (CT) of the chest and ultrasonography of the whole abdomen were performed, which did not show any evidence of metastasis.

Treatment and follow-up

Following post-histopathological confirmation from the biopsy, the right great toe was amputated with a 1 cm margin, and the sample was again sent for histopathological confirmation. The resected great toe was confirmed as invasive melanoma, specifically acral lentiginous melanoma. The Breslow depth of invasion was 2 mm, with a Clark level III classification, without ulceration and with negative margin status. The lymphovascular and perineural invasion was absent. The American Joint Committee on Cancer (AJCC) 8th Edition classifies it as pT2aNxMx. After the patient was discharged, he was kept under close follow-up, since he has a high risk of recurrence. The follow-up visit eight days post-discharge showed good wound healing. As a subsequent plan of action, the patient will be reviewed by the surgical and the medical oncologist every three months. Additionally, they will plan to undergo sentinel lymph node biopsy (SLNB) to see for any metastasis, along with serum lactate dehydrogenase (LDH) and serum S100B protein, which are considered to be raised in cases of metastatic melanoma and indicate poor prognosis of the disease. 

## Discussion

Subungual melanoma is a rare subtype of acral lentiginous melanoma, associated with poor prognosis, as 85% of cases are misdiagnosed during the time of diagnosis, with a reported five-year survival of only 27% [3]. Since the preliminary presentation of nail bed melanoma is brownish-black streak on the nail, it may often be misdiagnosed as benign conditions like onychomycosis, bacterial infection, subungual hematoma, pyogenic granuloma, subungual verruca, subungual exostosis, subungual hemorrhage, junctional nevi, glomus tumor, and malignant conditions like squamous cell carcinoma [3]. Further, it has been observed that tumor thickness is positively correlated with the likelihood of metastasis [4]. Discussing the treatment methods for patients with advanced melanomas, high-dose chemotherapeutic drugs like carboxamide, actinomycin-D, cisplatin, thiotepa, and interleukin-2 are used with minimal adverse effects. Adjuvant therapy is also important for treating advanced melanomas and preventing recurrences by usage of chemotherapy by single or multiple agents with recombinant interferon or IL-2 and radiation therapy. It is also seen that BRAF and NRAS mutations are seen in almost 80% of all sporadic melanomas, among which BRAF V600E mutations are considered the commonest. Multiple targeted therapies, such as vemurafenib, a BRAF inhibitor drug targeting BRAF V600E mutations, have become very crucial in the treating melanoma with increasing the life expectancy [4].

Clark and Breslow’s works are still relevant to date, and with tumor thickness, presence or absence of ulceration, mitotic rate, margins status, and presence of lympho-vascular invasion are being considered as the main prognostic factors for staging melanoma as per the recent AJCC melanoma staging system [6]. Further, early histopathological prognostication has led to the advances in the surgical management of melanoma, with the decision for early administration of chemotherapy, thereby improving the overall prognosis of the disease [6,7].

## Conclusions

The rarity of the presentation of subungual melanoma often leads to misdiagnosis and subsequent delay in appropriate management. Nail lesions in the elderly, or in the presence of any immune-compromised state like diabetes, should always raise the suspicion of subungual melanoma. A good incisional biopsy of the nail bed and matrix, followed by histopathological examination, is critical to establishing the diagnosis and can often improve the prognosis and rate of survival if performed in an early and timely manner, since tumor excision or amputation can often prove curative. To facilitate the timely diagnosis of such rare conditions, it is significant to foster a good collaboration between clinicians, oncopathologists, radiologists, medical oncologists, surgical oncologists, and other healthcare professionals.
